# Cohort profile: The Australian Paediatric Exposure to Radiation Cohort (Aust-PERC)

**DOI:** 10.1371/journal.pone.0271918

**Published:** 2022-09-09

**Authors:** Jasmine McBain-Miller, Katrina J. Scurrah, Zoe Brady, John D. Mathews

**Affiliations:** 1 Melbourne School of Population and Global Health, University of Melbourne, Carlton, Victoria, Australia; 2 Department of Radiology and Nuclear Medicine, Alfred Health, Melbourne, Victoria, Australia; University of South Carolina, UNITED STATES

## Abstract

Although the carcinogenic effects of high-dose radiation are well-established, the risks at low doses, such as from diagnostic X-rays, are less well understood. Children are susceptible to radiation induced cancers, and in the last decade, several cohort studies have reported increased cancer risks following computed tomography (CT) scans in childhood. However, cohort studies can be limited by insufficient follow-up, indication bias, reverse causation, or by lack of organ doses from CT scans or other exposures. Aust-PERC is a retrospective cohort designed to study the effects of low-dose medical radiation exposure, primarily from CT scans, in young Australians. The cohort was ascertained using deidentified billing records from patients who were aged 0–19 years while enrolled in Medicare (Australia’s universal healthcare system) between 1985 and 2005. All procedures billed to Medicare in this age/time window that involved low-dose radiation were identified, and persons without such procedures were flagged as unexposed. The Aust-PERC cohort has been linked, using confidential personal identifiers, to the Australian Cancer Database and the National Death Index, on two occasions (to Dec. 2007 and Dec. 2012) by the responsible government agency (Australian Institute of Health and Welfare). Deidentified Medicare service records of all radiological procedures including CT scans, nuclear medicine (NM) scans and fluoroscopy and plain X-ray procedures have been available to derive estimated radiation doses in the cohort. Records of other medical and surgical procedures, together with demographic and socioeconomic variables are being used in analyses to assess biases arising from reverse causation and confounding. After excluding patients with errant records, 11 802 846 persons remained in the baseline cohort, with an average follow-up time of 22.3 years to December 2012. There were 275 489 patients exposed to diagnostic nuclear medicine scans and 688 363 patients exposed to CT scans before age 20 and before cancer diagnosis. Between 1 January 1985 and 31 December 2012, there were 105 124 deaths and 103 505 incident cancers. Dose-response analyses based on the relevant organ doses are underway for individual cancers, and we plan to extend the follow-up for another 8 years to Dec 2020. Analyses using this very large Aust-PERC cohort, with extended follow-up, will help to resolve international uncertainties about the causal role of diagnostic medical radiation as a cause of cancer.

## Introduction

Recent studies have found that low-dose medical procedures in childhood are associated with increased cancer risks in later years [1–3]. However, while the association between high-dose radiation exposure and cancer is well-established, the causal significance of low-dose exposures from diagnostic imaging is still contested, particularly due to concerns of reverse causation and indication bias [4, 5]. Children seem more radiosensitive for some, albeit not all, cancer types; they are more susceptible to radiation-induced myelodysplasia, brain cancer, and thyroid cancer, but have lower susceptibility than adults to lung cancer [6]. Within Australia, the rate of federally funded CT scanning among patients aged 0–19 years more than doubled between the years 1985–2005 [7]. In more recent years, the rate of CT scanning among Australian children has decreased from 8.2 scans per 1000 children in 2008/09, to 6.1 scans in 2013/14 [8]. While the risks associated with exposure at an individual level may be small, the effects at a population level could be significant. However, when CT scanning is indicated on clinical grounds, these risks are more balanced by the medical benefit to the patient.

Medicare is Australia’s universal health care system, established in 1984. It registers all Australian residents across all states and territories, and provides full or partial payment to public and private healthcare providers who bill Medicare on a fee-for-service basis. Electronic records of services billed to Medicare are held by Services Australia, and the Australian Institute of Health and Welfare (AIHW) can access and extract these data for approved research projects. The Australian Paediatric Exposure to Radiation Cohort (Aust-PERC) uses the Medicare dataset and was established to assess cancer risks following low-dose diagnostic imaging radiation exposures, particularly from computed tomography (CT) scans during childhood or adolescence. However, as Medicare captures all relevant medical services, Aust-PERC has been able to capture other forms of radiation exposure, including nuclear medicine procedures, fluoroscopy, and radiotherapy.

## Materials and methods

The Aust-PERC study brings together information from three sources: the Medicare billing data, the Australian Cancer Database (ACD), and the National Death Index (NDI). Important variables from each data source are summarised in Table 1.

**Table 1:** Variables of interest measured across the three databases.

Medicare dataset (services data)	Australian Cancer Database	National Death Index
Date of birth (month and year)	Date of birth (month and year)	Date of birth (month and year)
Medicare item number	ICD-10 codes (including topography and histology codes)	Date of death (month and year)
State/territory where service was rendered	Date of diagnosis (month and year)	Cause of death
Date of service (DD/MM/YYYY)	State/territory where diagnosis was recorded	State/territory where death was recorded
Socio-Economic Indexes for Areas (a relative score of the socio-economic status of the patient’s postcode) [22]		
Sex (male or female)		
Date first known to Medicare (DD/MM/YYYY)		

### The Medicare billing data

Australians are enrolled in Medicare from 1984, from birth, or on becoming a permanent resident. A unique but confidential Medicare number allows funded services to be traced across time for each individual. For our cohort follow-up, Medicare records for each person were probabilistically linked to national cancer and death records held by AIHW, and de-identified by using a second number unique to each individual. Thus, this cohort is able to capture diagnostic imaging radiation procedures and radiation-attributable cancers across several decades.

Medicare service data until age 20 and outcomes were obtained for all Australians born between 1966 and 2005 who were enrolled into the Medicare system by 31/12/2005. Characteristics of the cohort are shown in Table 2. The cohort was de-identified to protect confidentiality, so that individual consent was not required; for research purposes, each individual record was linked using an anonymous but unique “patient number”. This meant all eligible individuals could be captured, ensuring a truly representative cohort. Eligible individuals entered the cohort on the last of the following dates: date of birth (month and year), date first known to Medicare, or 1 January 1985. This cohort does not contain individuals who enrolled in Medicare after their 20^th^ birthday, or after 2005.

**Table 2:** Characteristics of the Medicare cohort.

	Number (%)
**Sex**	
Male	5 973 555 (50.6)
Female	5 829 921 (49.4)
**Age (y) at cohort entry**	
0–4	6 739 812 (57.1)
5–9	1 562 883 (13.2)
10–14	1 818 859 (15.4)
15–19	1 681 292 (14.2)
**Age (y) at cohort exit**	
0–14	2 084 083 (17.7)
15–24	2 860 551 (24.2)
25–34	2 902 416 (24.6)
35+	3 955 796 (33.5)
***N* CT scan exposures**	
0	11 114 483 (94.2)
1	564 097 (4.8)
2–4	118 411 (1.0)
5–9	5271 (<1.0)
10+	584 (<1.0)
***N* diagnostic nuclear medicine exposures**	
0	11 527 357 (97.7)
1	206 346 (1.8)
2–4	62 216 (<1.0)
5–9	6076 (<1.0)
10+	851 (<1.0)
***N* exposed to both CT and nuclear medicine**	72 191
***N* other diagnostic radiology** ^a^	
0	5 401 217 (45.8)
1–4	4 687 179 (39.7)
5–9	1 323 419 (11.2)
10+	391 031 (3.3)

^a^ Excludes interventional radiology and UV therapy.

Whenever a Medicare funded service is rendered for an individual, an item number unique for the type of service rendered is recorded. These item numbers and their corresponding descriptors (available online through www.mbsonline.gov.au) were reviewed by researchers. Services Australia provides a list of broadly categorised services, including a “Diagnostic Imaging” category and “Radiotherapy and Therapeutic Nuclear Medicine” category, meaning relevant services involving radiation exposure could be easily identified. In this way, CT and nuclear medicine scans were extracted and grouped from more than 667 million billed services in the Medicare dataset [7, 9]. Medicare item code descriptors can change over time as new services are added, services are retired, or the definition of the service changes. All these changes are available through the Medicare Benefits Schedule website, so researchers were able to ensure services were correctly tracked across time.

### Probabilistic linkage to national outcome datasets

Using confidential personal information available through Medicare enrolment data, AIHW probabilistically linked first cancer diagnosis and death records to Medicare patients who were born after 1965 and who enrolled in Medicare in the years 1985–2005 and before age 20. Medicare records of services were initially linked to cancer and death records through to 31/12/2007; results have been reported elsewhere [10]. Subsequently, outcome follow-up has been extended for a further five years to 31/12/2012, with linkage to cancer and death records for all states and territories of Australia. As the probabilistic linkage was handled entirely independently of the Aust-PERC research team, details of the variables used to identify or exclude matches were not available to researchers. The ACD and NDI are both periodically updated, so that ICD-10 codes were provided to the researchers. To protect patient confidentiality, only the month and year of cancer or death were recorded; dates were rounded to the 15^th^ of the month for analyses.

### Retrospective organ dose estimation for CT scans exposures

The Medicare item descriptors, along with patient information, were used to retrospectively estimate organ doses from CT scan exposures based on population assessments [11]. In brief, CT scans were identified from Medicare items and grouped by scan type (i.e., body part scanned) [7]. The National Cancer Institute dosimetry system for CT (NCICT) [12] was used to calculate organ doses based on these CT scan categories, reconstructed technical parameters, year of scan, sex and the patient’s age at scan. Effective doses for nuclear medicine procedures have also been estimated based on typical administered radioactivity and isotope, year of scan, and the patient’s age at scan [9].

### Ethical considerations

This study has been approved by Human Research Ethics Committees of the University of Melbourne, AIHW, and by ethics committees and data custodians for all Australian states and territories. Due to the de-identified nature of the cohort, individual consent was not required.

## Results

### Study population

**Fig 1:**
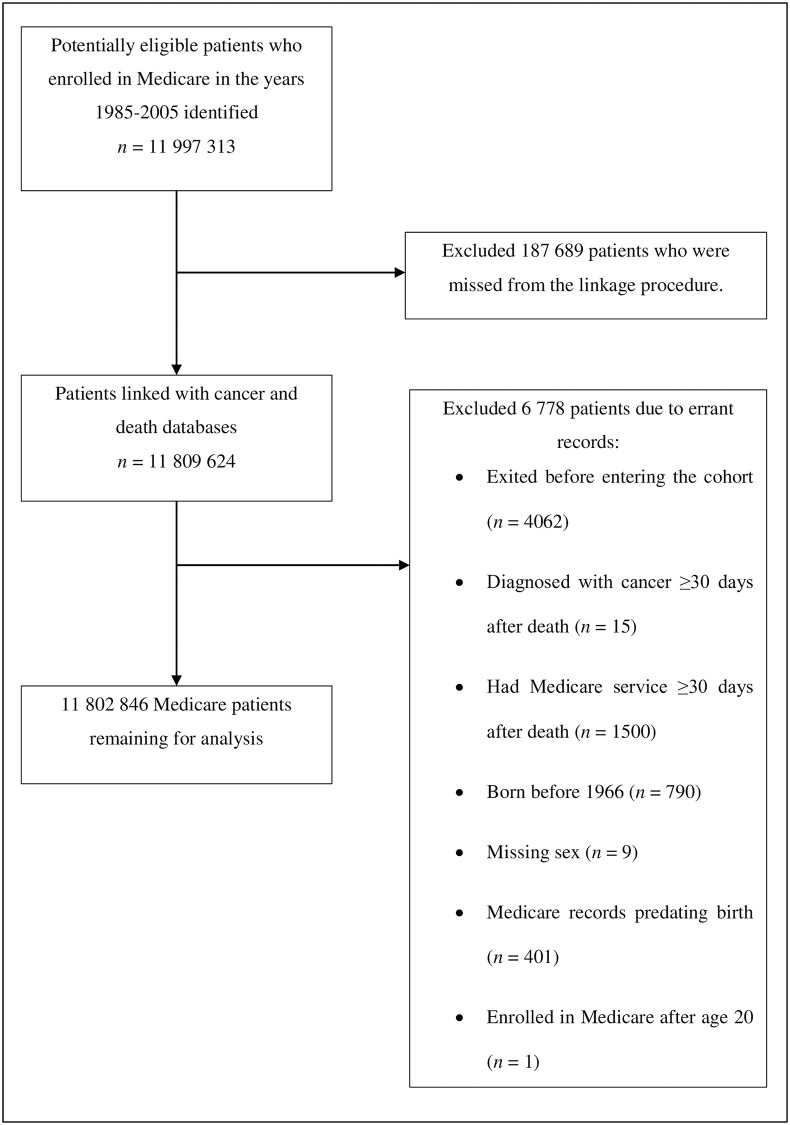
Flow diagram for the exclusion process used for the Aust-PERC study.

Fig 1 summarises the process for establishing the cohort. In total, 11 997 313 individuals who enrolled in Medicare before age 20 and before the end of 2005 were identified. 11 809 624 individuals were included in AIHW’s probabilistic linkage program. There were 187 689 individuals who had Medicare service records but were not included in AIHW’s linkage program. Given that any cancer diagnoses or deaths occurring among these individuals would not have been captured in our cohort, these individuals had to be excluded from the cohort. The vast majority of those excluded were born in 1965. The linkage program also missed some patients who were born towards the end of 2005, ostensibly due to a delay between actual Medicare enrolment and the date listed within AIHW datasets. A further 6778 patients were excluded either due to: 1) errors in their records, 2) the rounding of the outcome dates, meaning patients could die or be diagnosed with cancer before entering the cohort, or 3) because they were born in 1965 and were not consistently captured by the probabilistic linkage program.

Following the exclusions (Fig 1), 11 802 846 persons remained in the cohort, with an average follow-up time of 22.3 years. There were 275 489 patients exposed to diagnostic nuclear medicine scans and 688 363 patients exposed to CT scans before age 20 and before cancer diagnosis. As previously reported [7], the frequency of CT scanning within the cohort increased steadily over calendar time, before stabilising around the year 2000 (Table 3). Scans of the head were the most common type of CT scan within the cohort, though the proportion of scans targeting the head reduced from 80% to 63% between 1985 and 2005.

**Table 3:** Frequency of CT scan exposures, NM exposures, and other diagnostic procedures by year.

Year	CT scans performed	NM procedures	Other diagnostic procedures
1985	15 720	7927	818 071
1986	21 048	10 589	908 183
1987	23 195	14 099	934 079
1988	25 517	20 613	976 057
1989	27 151	23 355	979 688
1990	28 587	13 147	1 056 527
1991	29 725	14 510	1 062 610
1992	34 076	16 609	1 103 769
1993	36 361	17 950	1 146 295
1994	39 594	20 291	1 174 658
1995	41 799	21 675	1 170 653
1996	43 738	22 551	1 185 526
1997	46 662	23 029	1 196 157
1998	52 942	24 618	1 199 556
1999	56 120	24 807	1 227 742
2000	54 770	23 959	1 215 530
2001	59 849	24 094	1 246 533
2002	60 549	22 880	1 250 843
2003	60 796	21 043	1 218 594
2004	58 346	19 283	1 212 897
2005	61 544	19 273	1 230 888

### Incident cancers and deaths in Aust-PERC

**Fig 2:**
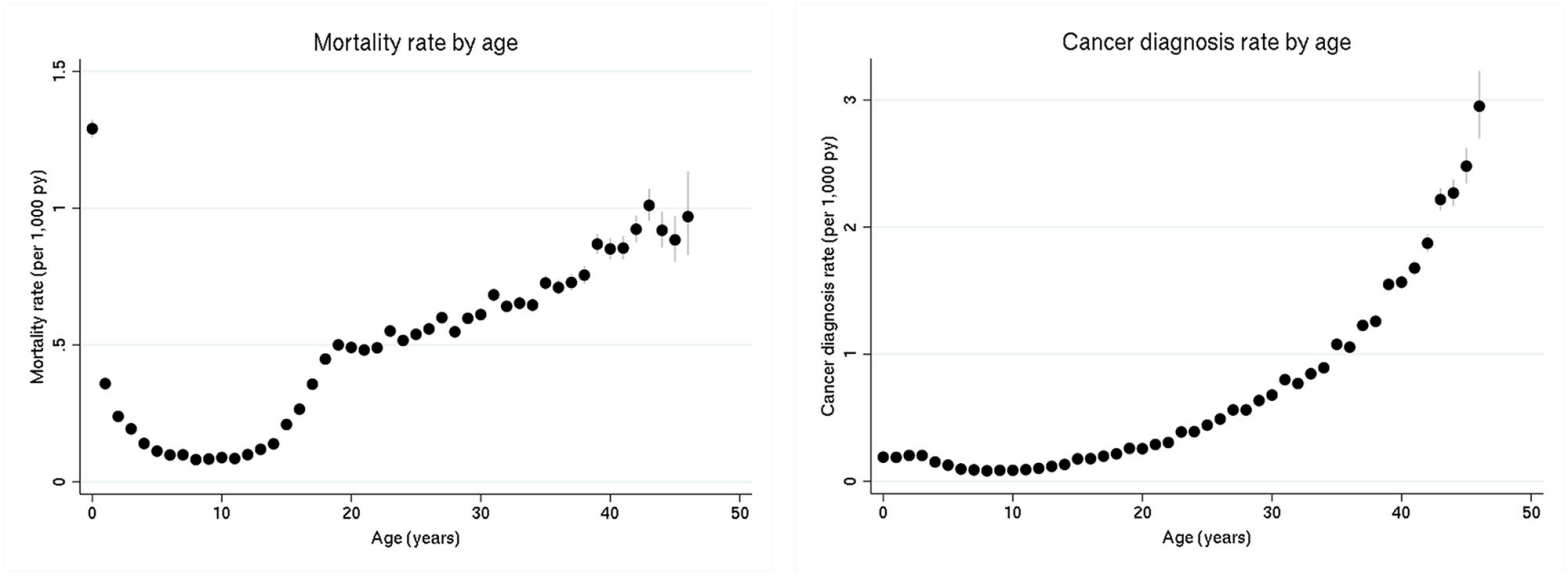
Rate of mortality (left) and cancer diagnosis (right) per 1000 person-years (py) for members of the cohort, by age. The dots represent the estimated rate, while the vertical grey lines represent the 95% confidence interval.

There were 105 124 deaths and 103 505 incident cancers (Table 4) linked to the cohort during follow-up to 2012. Mortality rates and cancer diagnosis rates varied by age (Fig 2). The childhood mortality rate was highest within the first year of life. Subsequently, the mortality rate dropped to a minimum around age 10, before increasing again. Cancer was rare during childhood, with ages 5–14 having the lowest rate of cancer diagnosis across our cohort. Cancer rates increased after age 20.

**Table 4:** Cancer diagnoses ^a^ in 1985–2012 among individuals in the Aust-PERC study.

Cancer type (ICD-10 codes)	Frequency			Total Percent
	Male	Female	Total	
Mouth and pharynx (C00-14)	2335	1029	3364	3.3
Digestive organs (C12-26)	3762	3645	7407	7.2
Respiratory organs (C30-39)	1056	823	1879	1.8
Bone (C40-41)	1103	758	1861	1.8
Melanoma (C43-44)	10 536	12 614	23 150	22.4
Soft tissue (C45-49)	1518	1152	2670	2.6
Breast (C50)	51	11 670	11 721	11.3
Genital organs (C51-58, C60-63)	7987	6036	14 023	12.5
Urinary tract (C64-C68)	1539	1120	2659	2.6
Brain (C69-72)	3596	2701	6297	6.1
Thyroid (C73-75)	1821	5689	7510	7.3
Ill-defined, secondary, unspecified (C76-80)	364	375	739	0.7
Myelodysplasias (D45-D46, D47.1, D47.3)	471	558	1029	1
Hodgkin lymphoma (C81)	2702	2389	5091	4.9
Other lymphoma (C82-83)	2438	1360	3798	3.7
Other lymphoid tumours C84-90)	1069	649	1718	1.7
Lymphoid leukaemia (C91)	3000	2118	5118	4.9
Myeloid leukaemia (C92)	1567	1252	2819	2.7
Other leukaemia (C93-96)	366	286	652	0.6
Total	47 281	56 224	103 505	100

^a^ Based on ICD-10 definitions.

### Publications and findings

In 2013, the first results from this cohort were published, with outcome follow-through to 31/12/2007 [10]. After accounting for age, sex, and year of birth, patients exposed to at least one CT scan before age 20 had a 24% greater risk of cancer than those with no CT scan exposure (incidence rate ratio = 1.24; 95% CI: 1.20, 1.29). Each additional exposure to CT scans before age 20 increased the relative risk by 0.16 (95% CI: 0.13 to 0.19). However, there were concerns regarding causality due to reasons such as sparse dose information, elevated risks of cancer despite short latency, and elevate cancer rates in sites not considered radiosensitive [4, 5].

More recently, finite mixture modelling was used to determine the length of time needed to separate brain cancers due to reverse causation from those that could be reasonably attributed to CT scan exposure [13]. The paper concluded that reverse causation bias was negligible when CT scans occurring less than two years before cancer diagnosis were excluded.

## Discussion

By probabilistically linking three Australian datasets, we have formed a large, nationally representative, retrospective cohort designed to estimate cancer risks following paediatric medical radiation exposure. In this paper, we provide an overview of the methodology used to establish the Aust-PERC, detail the inclusion and exclusion criteria, and provide a brief description of previous publications.

It is important to note that, while the Aust-PERC dataset contains exposures between the years 1985 and 2005 and before the patient’s 20^th^ birthday, cancer and death outcomes are followed through to end of 2012, regardless of age. This means many individuals are followed for outcomes many years beyond their exposure. The relationship between exposure and outcome could be confounded if there were variables related to both exposure and outcome. However, as most other cancer risk factors are likely to be independent of diagnostic exposures in early life, they would not be expected to confound the association between diagnostic radiation exposure and cancer outcomes.

### Strengths and limitations of Aust-PERC

Aust-PERC has a number of strengths. First, because of the large cohort size and long follow-up times, it delivers the statistical power needed to identify any radiation-attributable effects at low-doses and after latent periods which can be long. With an average follow-up time of 15.3 years from first CT scan and 15.5 years from first nuclear medicine exposure, there is sufficient time for radiation-attributable effects to be observed.

Another advantage of Aust-PERC is the organ dose estimation. Only a couple of past paediatric CT cohort studies have included a dose response analysis [3, 14, 15]. This is because the studies were retrospective in nature and lacked sufficient information to reconstruct doses. Observing a biological gradient, or dose-response, is an important aspect in determining whether an association is consistent with causation [16], and is therefore an advantage of Aust-PERC. However, these organ dose estimates were population-based and assigned the same dose to all exposures of the same type (body part) in individuals of the same age and sex for a given year; in other words, the doses could not take individual variation into account. Given that the Medicare Benefits Scheme (MBS) is available to all Australians, and registration with Medicare is close to 100% complete for Australian permanent residents, this study is representative of Australians born between 1966 and 2005. However, the MBS dataset did not contain useful demographic variables, such as weight, height, or ethnicity. Due to the size of the cohort and data anonymity, it would not have been possible to obtain these data.

The dependency on billing records for ascertainment of CT and nuclear medicine scans, rather than self-reported data, reduced bias as prospectively reported exposures are independent of outcome. However, the cohort does not capture those CT scans of cohort members that were not funded by Medicare under federal fee-for-service arrangements. Missing scans would include those in state-funded hospitals not billed to Medicare. The proportion of scans in state-based hospitals that were not funded by Medicare vary by state and decade. For the financial year 2008–2009 the percentage of paediatric CT scans occurring in state-based hospitals that were billed to Medicare varied from 7% in Western Australia to 44% in New South Wales [17].

We did not collect information on any scans before 1985 or after 2005. This will underestimate the total number of CT exposed patients. Furthermore, Aust-PERC does not contain CT or NM records beyond age 19, despite individuals being followed for outcomes well into adulthood. CT scan exposure rate increases with age [18, 19], so the absence of records for adult scans would underestimate the total number of exposures within the cohort, as well as underestimating the cumulative dose. While adult exposures were missing from our cohort, they were not considered to be a major concern for two reasons. First, children are more radiosensitive than adults and thus their exposures are more consequential. Second, due to the latency period of radiation-induced cancers, an exposure during adulthood would likely be closer to the end of follow-up and contribute less to the overall risk.

The Medicare database does not record the clinical indications for CT or nuclear medicine scans, which limits the potential to deal with confounding by indication. However, the dataset contains all Medicare funded services over the exposure period. Many of these services, such as a history of shunt insertion for hydrocephalus or multiple attendances with specialists, are indicative of underlying health conditions, and can be used to study indication bias or reverse causation. Such services are currently being used in a propensity score analysis [20], to predict the likelihood of CT scan exposure among cohort members and control for indication bias, and may be used to explore the association between Medicare-funded shunt insertion for hydrocephalus and associated CT scans.

Probabilistic linkage to cancer and death records may have missed some outcomes, therefore underestimating the total number of cancers and deaths within the cohort. Furthermore, information on the emigration from the cohort is missing, which would overestimate the number of person-years and underestimate the number of outcomes in the cohort when cancers and deaths occurred outside of Australia.

The limitations of the organ dose estimation are described in detail in Brady et al. [11]. In brief, organ doses were not recorded at the time of the scan and had to be retrospectively estimated. The accuracy of the estimated organ doses partly depends on the quality of the Medicare item descriptors, which were not intended for the purposes of dose reconstruction. Moreover, doses were estimated based on age, scan type, sex, and year of scan strata. Given that this does not account for variations in body size or scanning parameters, this procedure results in unmeasured variability in the form of Berkson error [21]. Despite these limitations, the Aust-PERC study remains the largest cohort of paediatric CT scans with organ doses in the world.

#### Ongoing work

Aust-PERC data are being used in innovative ways. For example, we have been able to interrogate comprehensive records of healthcare service use to predict CT scan exposure and to control for indication bias in propensity score analyses. We are also working on several dose-response analyses across multiple cancer types, and plan to extend follow-up for incident cancers and deaths through to 31 December 2020.

## Conclusion

Aust-PERC is a large, nationally representative, retrospective cohort study comprising more than 11 million individuals, with nearly 700,000 CT scan exposed individuals and nearly 300,000 patients exposed to nuclear medicine scans. The cohort has already generated several publications, and work continues on dose-response analyses and causal inference analyses with our extended follow-up.
